# Commotio Cordis Caused by Violence in China: Epidemiological Characteristics Detected at the Tongji Forensic Medical Center

**DOI:** 10.1097/MD.0000000000002315

**Published:** 2015-12-28

**Authors:** Jiao Mu, Zhenglian Chen, Xinshan Chen, Wei Lin, Hongmei Dong

**Affiliations:** From the Department of Forensic Medicine, Tongji Medical College, Huazhong University of Science and Technology, Wuhan (JM, ZC, XC, WL, HD), and Department of Pathology, Hebei North University, Zhangjiakou, Hebei, P.R. China (JM).

## Abstract

Commotio cordis (CC) is a recognized rare cause of sudden death in which an apparently minor blow to the chest causes ventricular fibrillation and cardiac arrest. CC diagnosis is still a challenge for forensic pathologists. A retrospective study of 9794 autopsy cases was conducted at the Department of Forensic Medicine, Tongji Medical College (DFM-TMC, China) from 1955 to 2014. A total of 39 cases (0.4%) were determined to be caused by CC. A male preponderance (male to female of 37:2) was found in the victims, whose age ranged from 13 to 47 years, including more than 85% individuals in their 10s and 20s. Most victims (27 cases, 69.2%) came from village. The highest rate of victims was found for middle school and college students (15 cases, 38.5%), followed by prisoners (11 cases, 28.2%), farmers (9 cases, 23.1%), workers (3 cases, 7.7%), and office staff (1 case, 2.6%). Chest blows were produced by fists (28 cases, 71.8%), feet (6 cases, 15.4%), knee (2 case, 5.1%), head (1 case, 2.6%), or objects (2 cases, 5.1%). Witness statements indicated that most victims collapsed after being impacted in the precordium. The autopsy findings were unremarkable except bruises, contusions, or subcutaneous hemorrhage in the anterior chest (13 cases), bleeding of intercostal muscles (5 cases), and disperse focal petechiae of the epicardium (11 cases). All CC cases in this study were caused by violent attacks and related to criminal processes. Correct diagnosis of CC due to violence has important implications in the judicial system.

## INTRODUCTION

Commotio cordis (CC) is a rare and fatal mechano-electric arrhythmogenic syndrome. Nesbitt et al^1^ defined it as “ a mechanical stimulation of the heart by nonpenetrating, impulse-like impact to the precordium that, through intrinsic cardiac mechanisms, which gives rise to disturbances of cardiac rhythm of various type, duration, and severity, including sudden cardiac death, in the absence of structural damage which would explain the observed effects.” In the middle of the 18th century, CC was firstly described in the context of chest trauma among workers due to accidents;^2,3^ then it was distinguished from contusio cordis by Schlomka.^4^ In recent years, CC has been increasingly reported and is considered an important cause of sudden cardiac death in young individuals mainly involved in sporting activities. Data from the National Commotio Cordis Registry in Minneapolis showed that 224 cases were reported from 1996 to 2010.^5^

It is now realized that CC events also can occur in violent attack, indeed sporadic accounts of CC caused by violent attack have been documented in forensic practice.^6–8^ However, this has not attracted widespread attention in the general public and even the medical community. Moreover, this kind of CC is very likely to enter the criminal justice system for murder prosecution. Thus, CC due to violent attack should be paid more attention in the forensic science. To our knowledge, relatively few detailed analytical data from autopsy studies on CC caused by violent attack have been published. The objective of this study was to describe the epidemiological characteristics of the 39 CC autopsy cases examined by the Department of Forensic Medicine, Tongji Medical College (DFM-TMC), Wuhan, China. Moreover, the pathogenetic mechanism, diagnosis difficulty, and forensic implications of CC due to violent attack were also discussed.

## MATERIALS AND METHODS

### Study Sample

As 1 of the 6 major forensic institutes in China, DFM-TMC assists police, courts, public health departments, hospitals, and sometimes the decedents’ families to provide forensic services such as autopsy, pathological examination, and toxicological analysis in central China. All autopsy cases assessed by DFM-TMC from January 1, 1955 to December 31, 2014 were reviewed. The causes of death were determined after complete and systematic autopsy, medical history review, suspect and witnesses’ testimony, and toxicological analysis. Age, gender, occupation, police investigation reports, and autopsy findings were analyzed.

According to the references, the inclusion criteria of CC was:^9,15^ verifiable documentation of the event occurrence; a witnessed event that victim collapsed instantly after a blunt blow to precordial area; no structural injury of the sternum, ribs, and heart; no underlying cardiovascular diseases. Excluded were cases with: severe tissue decomposition, lethal diseases or pathological changes, and positive toxicological evaluation.

Total 39 cases were diagnosed as CC. During the same period, 39 cases of sudden non-CC deaths, that matched the CC by age and gender, were selected as control group. The control group included 26 cases died of severe craniocerebral injury, 7 cases of electrothanasia, 4 cases of mechanical asphyxia, and 2 cases of high falling death.

### Statistical Analysis

Statistical analyses were conducted by SPSS 20.0. Continuous data are presented as mean ± standard deviation, whereas categorical variables were expressed as number and/or percentage. Independent samples *t*-test was performed to compare the means between two groups. *P* value < 0.05 was considered statistically significant.

## RESULTS

**FIGURE 1:**
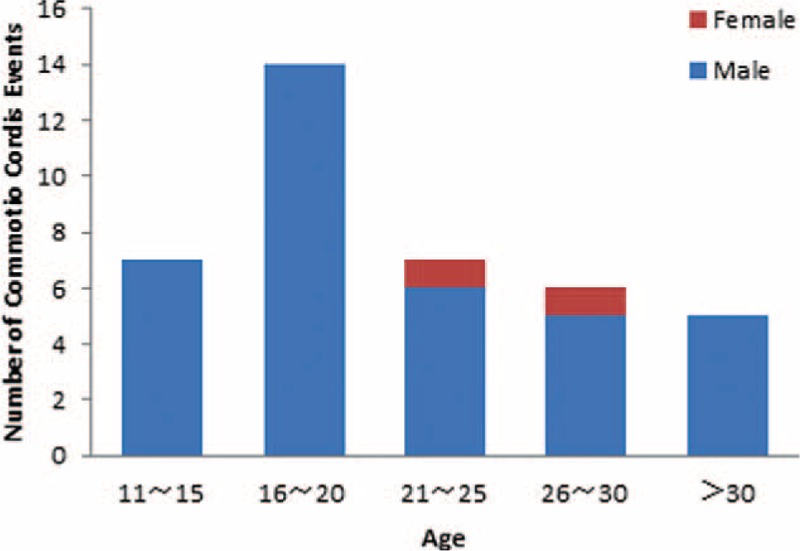
Distribution of commotio cordis events by age and sex.

**FIGURE 2:**
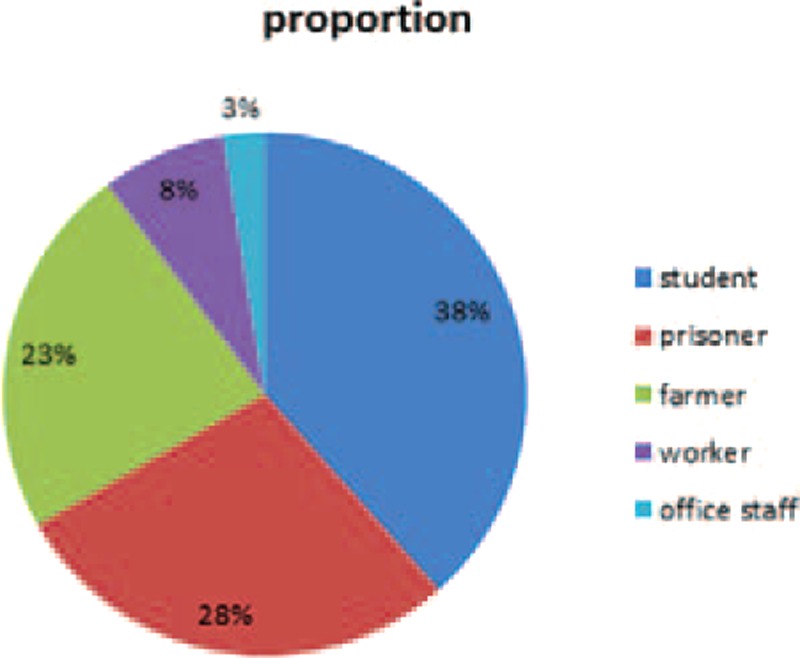
Distribution of commotio cordis events by occupation.

Between 1955 and 2014, a total of 9794 deaths were investigated by DFM-TMC, among which 39 cases (0.4 %) were determined to be caused by CC (Table 1). There were 37 males (94.9%) and 2 females (5.1%), aged between 13 and 47 years, including more than 85% individuals in their 10s and 20s (Figure 1). The highest rate of victims was found for middle school and college students (15 cases, 38%), followed by prisoners (11 cases, 28%), farmers (9 cases, 23%), workers (3 cases, 8%), and office staff (1 case, 3%) as shown in Figure 2. Accordingly, most cases occurred at school, home, in the prison or outdoors, and all were caused by fight or assaults. Chest blows were produced by fists (28 cases, 71.8%), feet (6 cases, 15.4%), knee (2 case, 5.1%), head (1 case, 2.6%), or objects (2 cases, 5.1%). Generally, the victims collapsed immediately after being impacted in the precordium. Some manifestations included pallor (9 cases, 23.1%), urinary incontinence (8 cases, 20.5 %), ptysis (7 cases, 18.0%), dyspnea (5 cases, 12.9%), and hyperspasmia (2 cases, 5.1%) (Table 2).

**TABLE 1:**
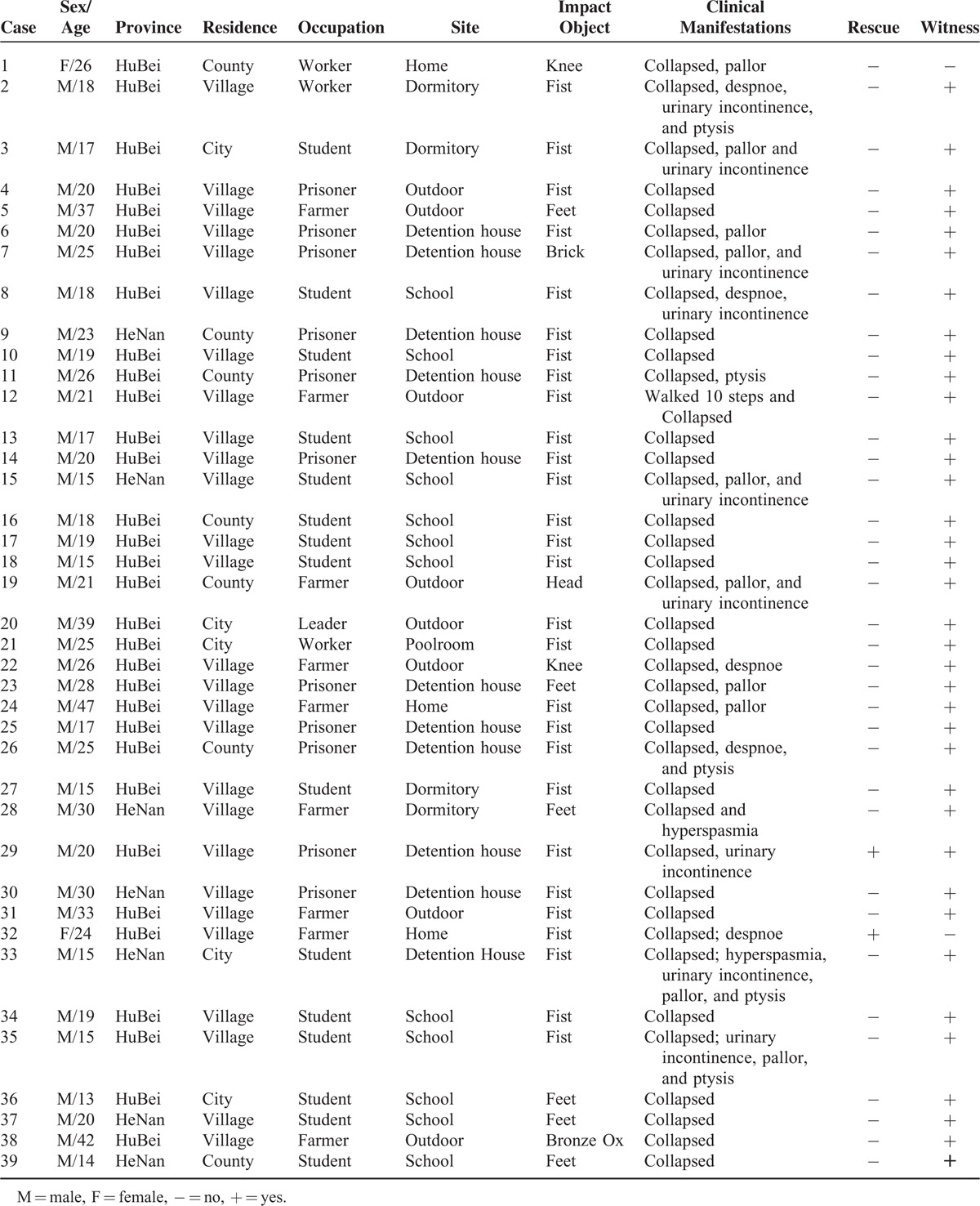
Summary of Cases

**TABLE 2:**
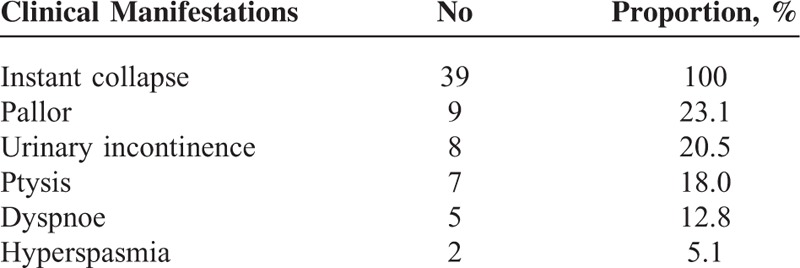
The Clinical Manifestations of Victims After Being Impacted in the Precordium

**FIGURE 3:**
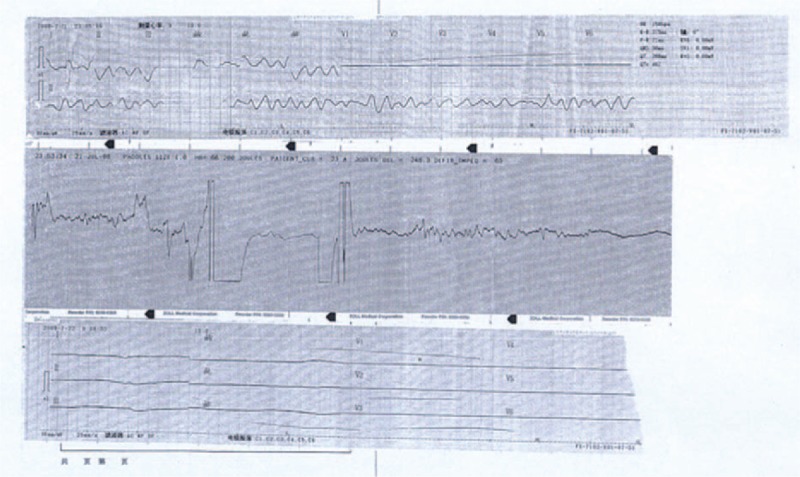
Electrocardiograms showing ventricular fibrillation.

Cardiopulmonary resuscitation was attempted only in 2 victims. In one case, the emergency medical services arrived on the scene within 15 minutes. Vital signs showed: heart rate, 30/minutes, blood pressure, 40/10 mmHg. In the second case, paramedics came 10 minutes after blow and found the victim in cardiac arrest. She was administered chest compressions immediately and electrocardiography showed ventricular fibrillation (Figure 3). Despite aggressive resuscitation, the victims finally died.

**FIGURE 4:**
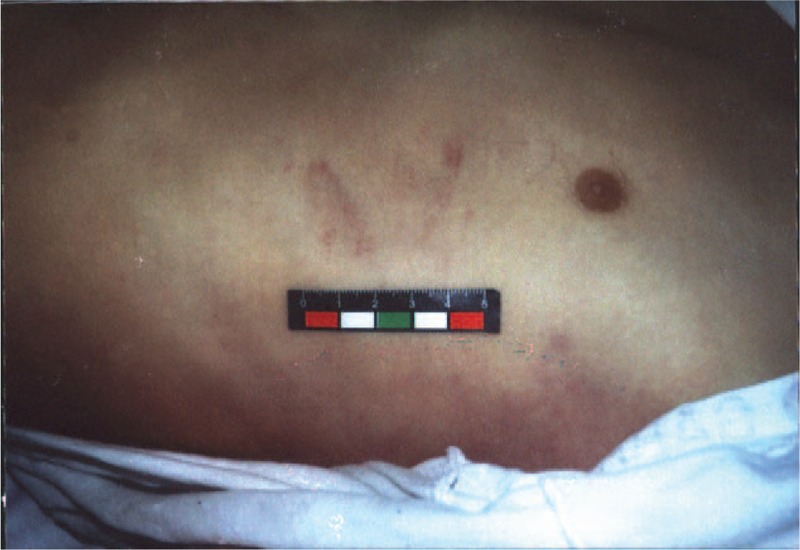
External examination showing bruise in the anterior chest.

The systematic autopsy and toxicological reports for each case were reviewed carefully (Table 3). The victims's average body mass index (BMI) was 21.14 ± 1.2, which was a little lower than that of the control group. However, the difference of BMI between the 2 groups is not significant (*P* > 0.05). Notably, all cases were witnessed or had documentation of the events leading to CC. Gross examination of the body revealed no remarkable finding except for bruise, contusion, or subcutaneous hemorrhage in the anterior side of the chest in 13 cases (33.3%) (Figure 4). Internal examination showed no injury of the sternum, ribs, lungs, heart, or main artery. There was intercostal muscle bleeding around the precordium in 5 cases (12.8%) and disperse equirotal petechiae of the epicardium in 10 cases (25.6%). The hearts were normal in size and 6 cases that showed relative heart hypertrophy (330–360 g). However, the difference of heart weight between the 2 groups is not significant (*P* > 0.05). No abnormalities were observed in the coronary arteries, cardiac valves, pericardium, or conduction system except for disperse focal petechiae of the epicardium in 11 cases (28.2%), and focal contraction band necrosis in 1 case. The remaining internal organs were normal macroscopically and microscopically. Postmortem toxicological analysis yielded negative results.

**TABLE 3:**
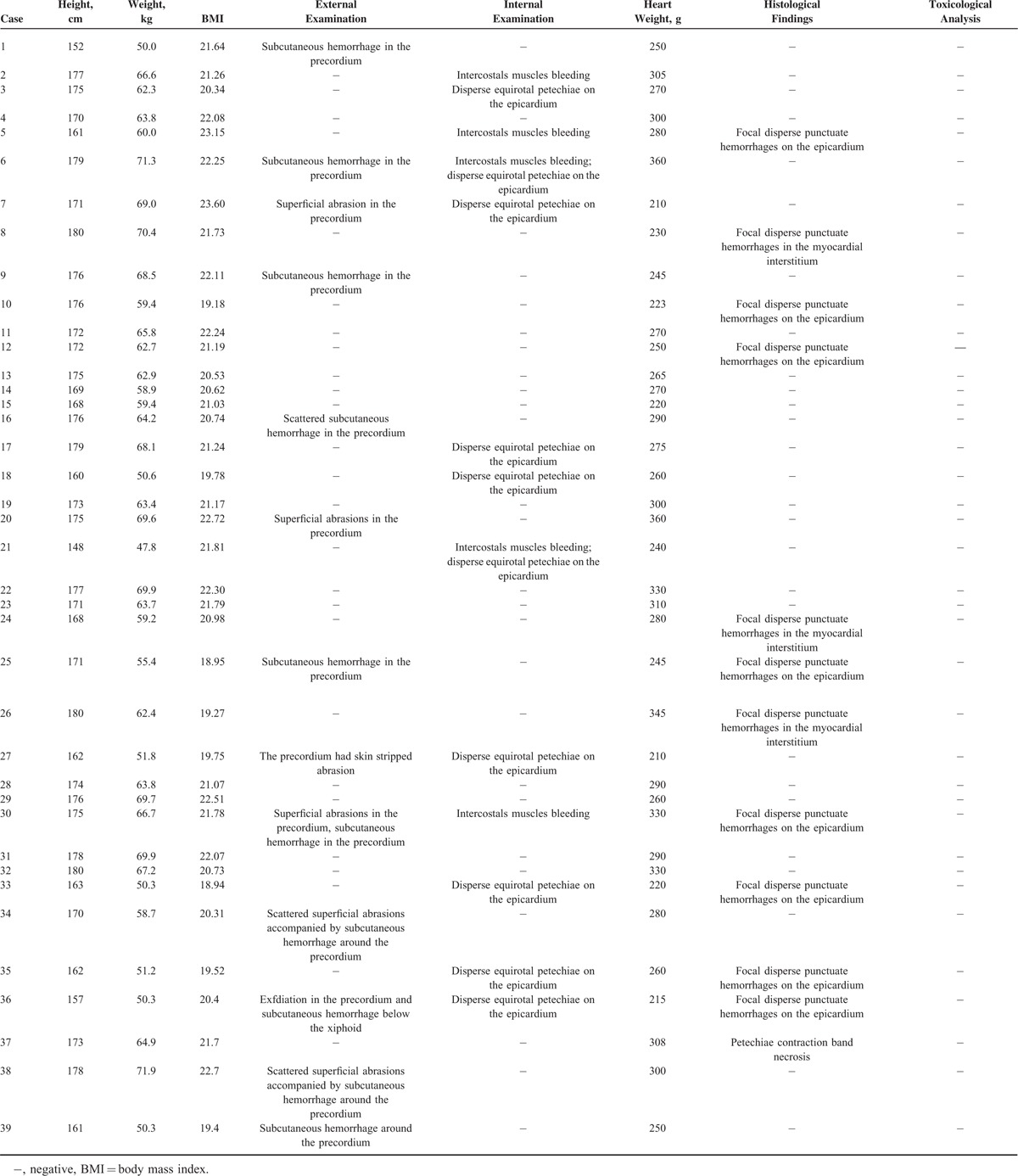
Autopsy, Histological Findings, and Toxicological Analysis

All victims came from central China including Hubei and Henan Province, of which 27 cases (69.2%) from village, 7 cases (18 %) from county, and 5 cases (12.8%) from city. A total of 14 of the 39 cases had the detailed police investigation about family information, with 5 cases that showed special family background: a middle school student lived with her grandma after her parents’ divorce; a high school student was a “left behind” children whose parents and 2 brothers were migrant workers and rarely at home; a technology high school student was raised by his mother since his father died, his mother lived by farming and had a lower education; and the other 2 female victims were all housewives and suffered from physical abuse by their intimate partner.

## DISCUSSION

This study presented the first-hand material regarding forensic examination of CC deaths in China by an independent institute. All the CC deaths were caused by violent attack, with many aspects in epidemiology differencing from findings described for US cases, where CC mostly occurs in sporting activities (Table 4). The preference in violent events may be explained in part by the typical American sports such as baseball and ice hockey, which are considered risk activities for CC in US, are not as popular in China. Currently, the precise incidence of CC resulting from assaults is still unknown because of the worldwide absence of systematic and mandatory reporting. On the basis of data from the National Commotio Cordis Registry in US, only 6 CC cases resulting from blow during fights and scuffles were found from 1991 to 2001 (0.6 cases per annum).^10^ We identified 39 cases of CC after reviewing 9794 autopsies during the past 60 years (0.65 cases per annum), the incidence is similar to that of US.

**TABLE 4:**
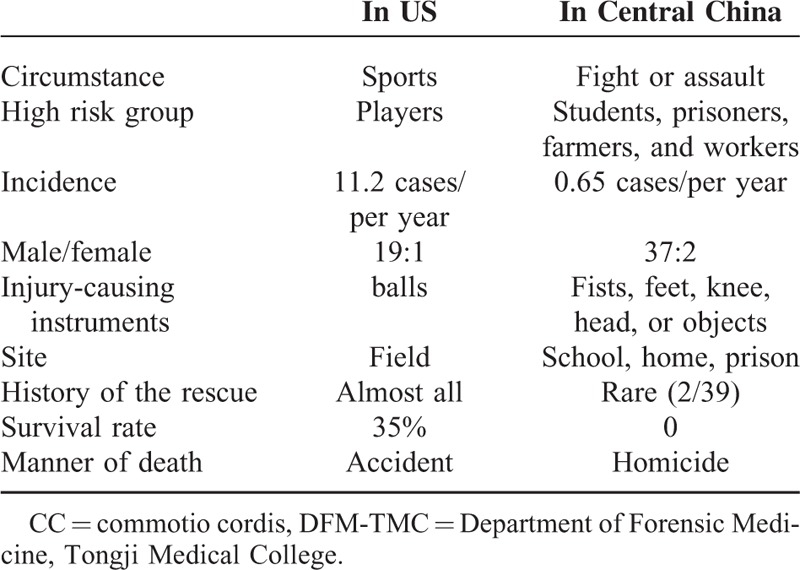
The Comparison of CC in Sports in US and CC Caused by Violence in DFM-TMC

As previously reported,^5,11^ most CC victims in this study were adolescents and young individuals. The higher prevalence of CC in individuals younger than 20 years is likely related to more ball-related sports and fighting participations. Moreover, that the relatively thin, underdeveloped, compliant chest cage (and immature intercostals musculature) in young people allows a more easier transmission of energy to the heart upon impact.^12,13^ However, the average BMI of the victim was not statistically different compared to the control group in our study. Notably, victims are overwhelmingly male. A plausible explanation for the male predominance is that they are more aggressive compared with females and more likely influenced by fight participation. Link et al^11^ speculated that differences in genes encoding ion channels and their biological modification by sex hormones may also be related to a higher incidence of CC in males. Unlike most US CC decedents who were players, the majority of victims were immature students and louche prisoners in the present study. Our findings indicate that these individuals might be a high risk group for CC occurring as a result of violent attack.

According to the current study, most victims came from village and born of a worker or farming family in which most of family member did not have a good economic situation and receive a good education. Family history showed some victims were “left behind” children or came from the single parent family. So, the special familial environment might affect the victim's psychological development, who are more irritable and aggressive. Therefore, inharmonious, poor socioeconomic family might be the social risk factors of CC due to violence.

In general, cardiovascular collapse is virtually instantaneous. However, approximately 20% victims remain physically active for a few seconds after the chest blows, which may display that different people have different tolerance with ventricular tachyarrhythmia.^5^ For example, a farmer being punched in the precordium was able to walk 10 steps before collapsing in one of the studied cases. CC is usually fatal.^14,15^ Mortality approaches 97% when resuscitative efforts are delayed longer than 3 minutes.^16^ Mortality is associated with the failure to initiate appropriate, aggressive, and timely measures of resuscitation by bystanders.^17^ In most US victims, efforts at cardiopulmonary resuscitation were initiated by bystanders or emergency medical technicians immediately or on time (within about three minutes), which resulted in higher survival rates of 35%.^9,18^ In stark contrast, the rescue rate was extremely low in our study. Therefore, it is important to enhance understanding and awareness of CC and popularize timely measures for cardiopulmonary resuscitation at the scene among the general public and the medical community in China.

The underlying mechanisms of CC were proposed as early as the 1930s.^19^ It is well known that location, timing, and energy of blows are important factors. Link et al^13,20^ demonstrated that impact must be directly over the heart (particularly at or near the center of the cardiac silhouette). Experimental data have shown that the impact should occur within a very narrow time window of vulnerability during repolarization, that is, 30 to 15 ms before the T-wave peak.^20,21^ The impact energy is not uniform, but closely related to the shape, size, hardness, and velocity of the impact object: harder, smaller, and sphere-shaped projectiles, such as baseball, softball, ice hockey, or lacrosse, are most likely to cause CC.^5,11^ High velocity impacts at about 64 km/hour are most likely to cause CC in experimental studies.^20^ Generally speaking, feet and fist are harder than various balls. Moreover, fist surface and tiptoe generate circular impacts with a small contact area. Fist is usually delivered at a speed of 60 km/hour, and the victims are often impacted repeatedly. Thus, blow from kick, fist, knee, and even stick could also trigger ventricular fibrillation in CC.^22^ The precordial bruises in autopsy represented the imprint of a blow location.

Currently, accurate diagnosis of CC is still difficult in forensic practice, because of the absence of a reliable objective pathological change. Serum biochemical analysis in CC is usually absent because most victims could not receive timely rescue and died before arriving at the hospital. The serodiagnosis and immunohistochemical studies are scattered and still only in study phase.^23^ Due to the lack of overt physical findings, an accurate diagnosis will rest heavily on witnesses and testimonies. In a witnessed assault, the association between the blow to the chest and the subsequent collapse is strongly suggestive of CC. To reach an accurate diagnosis, it is mandatory to know the death circumstances such as scene investigation, suspects and witnesses’ testimony, emergency medical records, and police reports. Also, complete and detailed autopsy is very important to exclude other pathological entities. In the present study, only 6 cases showed relative heart hypertrophy, which was not enough to explain the sudden death. In a word, forensic pathologists should be aware of the case circumstances and examine the precordium carefully, which may reveal injuries that could at least suggest the possibility of an impact.

It is worth noting that the sudden death due to congenital cardiac arrhythmia should be differentiated from actual CC cases. Unfortunately, antemortem electrocardiograms were not available to rule out rare congenital conditions. However, the victims were healthy without remarkable medical history, especially diseases of the cardiovascular system. Moreover, detailed analysis of the conduction system is a routine autopsy examination in such complicated and intractable cases since 1990 at DFM-TMC. Autopsy and histological examinations revealed no abnormality of the conduction system. Moreover, according to case investigation and postmortem autopsy, congenital arrhythmia was not a preferred cause of death.

Considering that the present cases could be related to criminal processes, correct diagnosis of CC has implications in legal proceedings. Meanwhile, from a medico-legal view, it is also important to judge the manner of death accurately. A few legal characteristics of CC caused by violence in general should be considered for identifying the manner of death: all CC occurrences followed intentional conflicts; chest blows were all delivered in a moment of anger, with varying degrees of force; all defendants had no awareness that a nonpenetrating blow to the chest of healthy people is inherently life-threatening, and could result in criminal liability for causing death; and all perpetrators did not expect the victim's death. Although cases were under different circumstances, the manner of death in all of them should be determined as a homicide.

According to medical literature, several similar CC cases have entered into the criminal justice system as subject of prosecution for murder.^10^ Due to misconceptions of CC within different criminal justice system, the conviction and measure of penalty are inconsistent.^10^ Although most defendants are prosecuted for murder, Lucena et al^24^ proposed that intentional homicide should be excluded under such circumstances. In reality, the occurrence likelihood of a CC event is not explained by the magnitude of force, rather its location and timing. The suspects do not predict the consequence when to administer the blow in a voluntary way. So, under such circumstances, homicide by imprudence is more proper.

## LIMITATIONS

Our study cannot completely reflect the precise incidence of CC resulting from assaults in China. The following aspects should be considered for the incidence of CC due to violence in our study: First, the autopsy rate in China is relatively lower compared with other developed countries because of the Chinese custom. Generally, an anatomy are only required and agreed by the relatives of the deceased in homicide or contentious cases. Second, a large number of cases with definite death are always handled by legal examiners in local police stations. The medico-legal examination in our department mainly focuses on complicated and intractable cases, such as suspicious deaths, unexpected deaths, and deaths related to medical and personnel dispute. Thus, the precise incidence of CC in China might be much lower than that of the present study.

## CONCLUSION

CC is a rare cause of death in forensic investigation, especially related to violence. This study showed a different epidemiological characteristic of CC in China. All CC cases assessed here resulted from assaults, fights, or domestic violence, which are related to criminal processes. So, correct diagnosis of CC is important for judicial fairness, especially for conviction and penalty measure. Based on its characteristics of emotional and intentional injury, and death unpredictability, the manner of death in CC should be considered homicide by imprudence. Meanwhile, it is important for forensic pathologists to help magistrates and police fully understand how a minor blow to the chest may result in the death of a young and healthy person. Moreover, the study also emphasized that people should be aware of the importance of avoiding precordial blow, and early recognition and timely rescue measures might increase the survival rate of CC.

### Ethical Standards

The authors declare that the study comply with the current laws of the country in which they were performed.
